# A New Apparatus for the Extension of Flexed Knee Joint

**Published:** 1859-03

**Authors:** 

**Affiliations:** Health Commissioner of Berlin


### A NEW APPARATUS FOR THE EXTENSION OF FLEXED KNEE JOINT.

BY DR. EULINBURG, HEALTH COMMISSIONER OF BERLIN.

An Address at a Meeting of the Association of Berlin Physicians, June 30, 1858. From the "Allgemeine Medicinische Central Zeitung."

The important progress which has been made since the time of Stromeyer in the treatment of articular contractions, is well known. By means of manual force (Brisement force alone), without previous division of tendons, we now reduce joints instantaneously, which have remained flexed for years. Yet cases have presented themselves to me, and, as we all know, to other physicians, in which this forcible extension has not terminated favorably. We have occasionally, with the consent of skillful colleagues, used our utmost strength, without gaining more than the simple laceration of the fibrous thread or cord-like tissue, produced around the bony structures of the joints, by the morbid processes of disease, which materially limit the degree of motion. In these cases, however, by the application of "Brisement force," there was always marked improvement. Mindful of the frequent and satisfactory results of this treatment in cases of apparent ankylosis vera, I am far from applying to it the epithet, "true work of an executioner," as given by some surgeons, especially by Lorinser, and am far from considering it as never justifiable.

On the contrary, I gladly employ this treatment in suitable cases with energy, and yet with due prudence, in order to attain our object without any possible injury. Any one of course would use care, who has once experienced unfortunate results; among which, though by no means the most unpleasant, I will mention a simple fracture above or below the ankylosed joint, as it happened to me in my practice some years since.

We have accounts of much worse accidents, in the practice of Louvrier for instance (Bonnet, Traite de therapeutique des maladies articulaires, Paris, p. 395), in which comminuted fractures and ruptures of the vessels and nerves occurred, which rendered amputation necessary, and which, without amputation, occasionally resulted in death.

Under such circumstances, I am of the opinion that there are cases of anchylosis in which, 1st, Brisement force cannot be employed with good results; and 2d, in which it ought not to be employed at all. In respect to the first category, I will only refer to cases of enlargement of the osseous portions of the joints, which is often an insurmountable objection to violent and complete extension. I frankly acknowledge, that, in such cases, when I have thoroughly reduced the joints, under the full influence of chloroform, I have often been obliged, in consequence of subsequent inflammatory action, to relinquish the advantage gained during anæsthesia, to combat the effects of the violent operation. On the other hand, we should not press with too much violence this treatment in cases in which the original disease still exists, or has only apparently run its course, and in which the new injury is in danger of rekindling the slumbering disease.

In such cases, gradual force is indicated, both in the commencement, and also as termination of the treatment after "Brisement force."

Since the introduction of the "endless screw" (worm), we have possessed an excellent apparatus for gradual extension, and yet I have not always found the apparatus adapted to every indication. There was still wanting an apparatus, which, with the advantages of an easy, regular, and gradually increasing power, would be competent to overcome the resistance caused by the ankylosis.

I believe that the improvements which were demanded by the old forms of apparatus, are found in my own. Instead of the two tooth wheels, which, in the common apparatus of Stromeyer, were set in motion by a key attached to an "endless screw," and instead of a single screw, running lengthwise, which, in Lorinser's apparatus, and that of Palasciano and Bonnet, was also set in motion by a key, I have used one spindle (or long screw) on each side. I have united these two spindles, near the joint, by means of a shaft, to which are attached a couple of "endless screws" (playing into the threads of the spindles), and turned by a crank. By turning this crank, the spindles cause the two portions of the apparatus, to which are attached the diseased limb, by means of padded splints, either to bend or extend at the will of the operator. In order that the apparatus may be used for either limb, I have made the crank movable, so that it can be applied to either side of the shaft, to suit the patient's convenience.

The good qualities of this apparatus are:

1st. The extraordinary ease of manipulation in attaining the desired object.

2d. The equality and perfect command over the speed of its motions.

3d. Its almost unlimited power.

4th. Its equally advantageous application for flexing a limb, ankylosed in an extended position.

5th. The application of the apparatus in producing passive flexion and extension of a limb already rendered movable, in which case the apparatus supplies gentle force in addition to the voluntary force of the patient himself.

6th. The use of the apparatus by the patient himself, either with or without assistance.

7th. The ready construction of the same into a portable apparatus, which cannot be said of Lorinser's or Palasciano's and Bonnet's apparatus.

For these reasons this apparatus is to be used with benefit:

1st. For treatment of flexed joints and straight ankylosis. In cases of the latter affections the old apparatus was wholly inapplicable. "Brisement force," however, I consider as contradicted in cases of ankylosis of the extended limb, since the subsequent inflammation may easily induce permanent flexion, which is really a still worse condition.

2d. For subsequent passive motions, which, in maintaining the advantage already gained by the extension, is of great benefit in keeping up flexibility of the joint. By this means we prevent a new ankylosis.

3d. For prevention of contractions and ankylosis in all morbid processes, in which such results are to be feared.

4th. For applying "Brisement force" (patient being under the influence of chloroform) in such cases in which the strength of the operator is insufficient, or in which there is danger of fracture in a wrong place, which, with this apparatus, cannot easily happen, since the fulcrum is exactly at the joint, and the force is applied equally to the whole limb.

Finally, I would say that the principles of this apparatus are applicable to contractions and ankylosis of all other joints, especially of the elbow, to which we formerly applied other forms of apparatus.

